# Defending explicability as a principle for the ethics of artificial intelligence in medicine

**DOI:** 10.1007/s11019-023-10175-7

**Published:** 2023-08-29

**Authors:** Jonathan Adams

**Affiliations:** Centre for Medical Ethics, Institute of Health and Society, Faculty of Medicine, University of Oslo, Kirkeveien 166, Fredrik Holsts hus, Oslo, 0450 Norway

**Keywords:** Explicability, Artificial intelligence, Medical ethics, Principlism

## Abstract

The difficulty of explaining the outputs of artificial intelligence (AI) models and what has led to them is a notorious ethical problem wherever these technologies are applied, including in the medical domain, and one that has no obvious solution. This paper examines the proposal, made by Luciano Floridi and colleagues, to include a new ‘principle of explicability’ alongside the traditional four principles of bioethics that make up the theory of ‘principlism’. It specifically responds to a recent set of criticisms that challenge the supposed need for such a principle to perform an enabling role in relation to the traditional four principles and therefore suggest that these four are sufficient without the addition of explicability. The paper challenges the critics’ premise that explicability cannot be an ethical principle like the classic four because it is explicitly subordinate to them. It argues instead that principlism in its original formulation locates the justification for ethical principles in a midlevel position such that they mediate between the most general moral norms and the contextual requirements of medicine. This conception of an ethical principle then provides a mold for an approach to explicability on which it functions as an enabling principle that unifies technical/epistemic demands on AI and the requirements of high-level ethical theories. The paper finishes by anticipating an objection that decision-making by clinicians and AI fall equally, but implausibly, under the principle of explicability’s scope, which it rejects on the grounds that human decisions, unlike AI’s, can be explained by their social environments.

## Introduction

Luciano Floridi and colleagues have suggested that the ethical implications of artificial intelligence (AI) across society can be analyzed in line with the popular principlist approach in bioethics, albeit with the notable addition of a principle of *explicability* (Floridi and Cowls 2019; Floridi et al. 2018). Thus, as well as adhering to Beauchamp and Childress’s (1979) principles of beneficence (doing good), non-maleficence (avoiding harm), respect for autonomy (as a facility for decision-making) and justice (in distributing benefits and harms), a doctor ought to use AI models only if they are explicable. But the principle of explicability’s addition has been controversial, with some critics arguing that, at least in principlism’s original territory of medicine, its requirements can be reduced to those of the traditional four principles of bioethics. This paper aims to defend the principle of explicability against such objections and thereby support its addition to the classic principlist account for the purposes of tackling the ethical implications of medical AI.^1^

After outlining an illustrative case of AI in medicine, which invites consideration of why similarly opaque AI models may be problematic, I describe the approach taken by Floridi and colleagues and what they think the principle of explicability brings to bioethical models. I then consider what I call the ‘reductionist’ critique of their five-principle framework and explain how it challenges the idea that a principle of explicability is needed, as these proponents suggest, to perform an enabling role in combination with the four classic principles. I argue that the critics’ argument rests on a mistaken assumption that the justification for a moral principle must lie at the most general normative level, which is the premise supporting their belief that the notion of an ‘enabling principle’ is inherently flawed. I suggest that, in keeping with the principlist tradition, explicability may plausibly derive its status as a principle instead from its mediating role between abstract theory and concrete decision-making and draw a comparison with the ‘mutuality principle’ already proposed in bioethics. On behalf of the skeptical ‘four principlist’, I consider how one might draw a parallel between the inexplicability of AI decision-making and that of ordinary clinicians to suggest that the principle of explicability either overreaches by making novel demands on the whole of medicine or rests on a double standard. In response, I argue that there is a disanalogy between human and AI decision-makers as the former are rooted in scrutable social structures, giving us explanations that some would reject but only by demanding that reasons be provided by decision-makers themselves, which merely imposes a new double standard in need of justification. Based on my defense, I conclude that five principles are indeed better than four when it comes to building an ethical framework for the development and implementation of AI in medicine.

## The basic problem of explanation in AI-driven medicine

Suppose you are a hospital doctor responsible for fielding admissions of patients with pneumonia. A key task is to decide which patients should be admitted to the hospital and which to treat instead as outpatients. You intend to base this decision in each case on an estimate of the patient’s risk of dying but voice your doubts about the accuracy with which you, or indeed anyone working at the hospital, can do so. Someone in your team comes to your aid and presents you with a novel tool: a machine-learning model that will predict the probability of a patient’s death based on a wider set of cases (its ‘training data’) than you could hope to carry in your head. The model contains a key flaw, however, having learned from its training data that patients with asthma are, all other things being equal, at a lower risk of dying from pneumonia. This defies all conventional wisdom, but there is a logic behind it: the likelihood of dying was indeed lower for patients with pneumonia in the group used as training data. The problem is that all other things are not equal in this case. Patients with pneumonia in the model’s dataset only fared better because they were admitted to intensive care for especially aggressive treatment that lowered their risk of death below the typical patient’s. The inference from asthma to a lower probability of death cannot, therefore, be validly applied in your case, where a patient’s risk before even being sorted into outpatient or inpatient categories is desired as the model’s output.

This is not a real case of AI being applied in medicine but rather a hypothetical exploration of a much-cited model that Caruana et al. (2015) revisit. The authors aim in their paper to improve the earlier model, but if offered to you as described above, why would your use of the model, from an ethical perspective, be problematic? One obvious reason is that it would be bad for anyone with asthma presenting to your hospital with pneumonia, who would be treated as outpatients and therefore unlikely, in an unfortunate twist of fate, to receive the very kind of aggressive therapy that skewed the prediction model’s training data. This aspect of the model may be superficial, however, given that with some knowledge of the respiratory system, one could easily spot that something was awry and use technical means, such as re-training the model on a new dataset, to rectify it. The deeper problem with the model, indeed the one identified by its original developers, is that there could be any number of issues beneath the model’s surface that would make it unsuitable for clinical application. If you proceeded to use the model in your work as a hospital doctor despite knowing this, it seems you would be acting irresponsibly or reprehensibly in some other way not captured by simply noting the anticipatable outcomes for patients with asthma.

In recent years, researchers have congregated around the notion of ‘explainability’ to describe what is desirable in AI models, typically those using a machine-learning approach, and lacked in the example already described. The concept of explainability is variously described, with some commentators applying a techno-scientific gloss on the concept of explainability and others a more political or moral one. Thus, Floridi and Cowls (2019, 8) suggest that their idea of ‘explicability’, explained more thoroughly in what follows, is helpful for “incorporating both the epistemological sense of ‘intelligibility’ (as an answer to the question ‘how does it work?’) and in [sic] the ethical sense of ‘accountability’ (as an answer to the question ‘who is responsible for the way it works?’)”. Both ideals have long histories in the AI ethics literature, especially where it overlaps with what Mitcham (1994, 137) identifies as the two schools of philosophy of technology: “engineering philosophy of technology” and “humanities philosophy of technology”. As Adamson (2022, 5) says, in the context of artificial intelligence, these schools capture respectively “the technical functioning of technology” as well as “larger questions, such as whether a technology should be adopted”. In technical literature, classically scientific notions of ‘transparency’ and ‘intelligibility’ have prevailed, with vast arrays of methods being designed to achieve and realize them through the design of models or post hoc explanations of their functioning (Lipton 2018). In the humanities, meanwhile, well-known ethical and political formulae have found a new lease of life in the ‘accountable AI’ discussions of late, with Maclure (2021) grounding the need for explainability in John Rawls’s notion of public reason, for example.

## Explicability explained

The idea of explicability, under various terminological guises and in epistemic and ethical forms, clearly enjoys widespread support and functions as an “umbrella term” (Ursin et al. 2023, 173) to encompass a vast array of concepts used in ethics and computer science. Thus, where Floridi and Cowls adopt a bifurcated conception of explicability, one can also conceive of explicability as encompassing elements beyond accountability and intelligibility to engage “other acts of communication and disclosure” (Jobin et al. 2019, 391) that have been linked to transparency. Ursin et al. (2023, 183), for example, identify “disclosure, intelligibility, interpretability, and explainability” as distinct levels of explicability. What makes the approach of Floridi and his colleagues stand out more than this restriction, however, is their novel presentation of explicability as a ‘principle’, which this paper focuses on unpacking in the context of these authors’ thinking and its prior usage in bioethics. Floridi et al. (2018, 696) suggest that Beauchamp and Childress’s four classic principles of beneficence, non-maleficence, autonomy, and justice can be applied fruitfully to their target field of digital ethics but also that this “should not be surprising”. Bioethics and digital ethics are, they say, united “in dealing ecologically with new forms of agents, patients, and environments” (Floridi et al. 2018, 696), echoing Floridi’s own long-held appeal for a “new ecological ethics for the information environment” (2002, 41). Still, though, Floridi et al. suggest that these familiar principles of bioethics cannot be carried over effortlessly to yield a thoroughgoing ethics of AI. Rather, “one more, new principle is needed in addition” (2018, 696), which is—as already indicated—explicability. By analyzing the place of the five combined principles in a range of recommendations from academia, political institutions, and nonprofit initiatives, Floridi et al. attempt to show their relevance to issues around AI ethics and the good of society at large. Though the authors only make use of the medical context as a comparator for the purposes of designing ethical standards, it is a clearly important subset of the societal contexts within which we find “AI systems that make socially significant decisions” (Floridi et al. 2018, 702).

As for the four classic bioethical principles themselves, Floridi et al. suggest that the principle of beneficence finds expression in the broad notion of AI being “beneficial to humanity” (Floridi et al. 2018, 696), under which some organizations include the protection of the planet on which humanity thrives. Meanwhile, the authors find that the principle of non-maleficence is invoked in injunctions against infringements of privacy and other irresponsible uses of AI, noting that the relevant duties of non-maleficence may apply to “accidental” and “deliberate” harms (Floridi et al. 2018, 697). The principle of autonomy arises, within the recommendations they review, in the face of threats not only to the capacity to make particular decisions, as is common in medicine, but also to “the power to *decide which decisions to take*” (Floridi et al. 2018, 698), rather than letting AI do so.^2^ Finally, justice appears in Floridi et al.’s analyzed recommendations in relation to risks around “unfair discrimination”, the distribution of benefits, and outcomes such as threatening “existing social structures” (Floridi et al. 2018, 699). The applicability of the four familiar principles of bioethics to AI does not appear to be in dispute; thus, the present paper focuses on the putative ‘principle’ of explicability and whether it ought to be considered as such. But it is important to note that Floridi et al. do not intend explicability to function as a principle in quite the same way as the standard four from bioethics (Morley et al. 2020). They see it as playing an enabling role such that the four core principles can be achieved, referred to as “the crucial missing piece of the AI ethics jigsaw” by Floridi and Cowls (2019, 8). As a second-order principle, explicability’s value hinges on its epistemic and ethical benefits, allowing us to understand the extent to which uses of AI models meet the standards set by the other principles and hold agents accountable when they do not.

To see how this applies to medicine specifically, consider the above example of an AI model used for managing hospital admissions. The case against such a model that would flow from Floridi et al.’s principle of explicability is twofold. Through an epistemological lens, one observes that in this case there is a realm of knowledge (about the logic underlying the model’s probabilistic judgements) that is inaccessible to those involved in the relevant decisions. This says something about *what* is lacking because, at least on one understanding of explanation, without “epistemic access either to the inner workings of x or to the complete causal or probabilistic context that determines the properties of the *explanandum* … it would be impossible to reach true explanatory information about x” (Páez 2020, 445). In this case, explaining why one patient was admitted and another was not will be difficult in at least some cases, even if one has a general knowledge of what the model was designed to do. The individuals for *whom* epistemic access is limited may be involved in their capacity as “decision-makers” or “decision-recipients”, as the standard distinction in the literature puts it (Information Commissioner’s Office and Alan Turing Institute 2020). The patient in the case we are imagining has no sway over admissions, but in other cases she could be both the recipient and the maker of a decision that suffers epistemically due to an opaque AI model. Meanwhile, from an ethical perspective, the model’s opacity would present a barrier to holding any relevant individuals or organizations accountable if there were systematic neglect of certain patients. Such an outcome may occur to clinically vulnerable groups, like the asthmatics who were more transparently affected but also socially disadvantaged groups that are subject to systemic biases, such as women and people of color (American Civil Liberties Union 2023). Failing to provide plausible routes to accountability, therefore, inexplicable AI models make it difficult to maintain ethical responsibility in either a backward- or a forward-looking sense, e.g., through retributive consequences for culpable individuals or mitigation of future risks (Hedlund and Persson 2022).

## The reductionist critique

The expansion of Beauchamp and Childress’s set of bioethical principles to include explicability has been welcomed by many, even being adopted by the European Commission’s Independent High-Level Expert Group on Artificial Intelligence in its ‘Ethics Guidelines on Trustworthy AI’ (European Commission Directorate-General for Technology 2019). In fact, where scholars have criticized Floridi et al.’s model, it has at times been on the grounds that *more* principles for AI should be added to the standard four from bioethics to capture multiple desiderata that Floridi et al. include under the category of explicability. Loi et al. (2020, 44), for example, contend that the epistemological and ethical aspects that Floridi and colleagues consider, along with the special value of human control over AI, merit *three* principles in addition to the standard bioethical four: “control, transparency, and accountability”. Given the previous section’s discussion of potential objectives that can be categorized under explicability, one can clearly see how some would view the addition of a single further principle as simplistic. Others, however, have responded to the five-principle approach by arguing that the addition of explicability to the four classic principles is in fact overcomplicated as a model of AI ethics. Since such a critique places a lid on the addition of any principles along the lines of explicability, I devote this paper to tackling its claims rather than assessing *more* expansive sets of principles, which can be proposed only on the assumption that some addition is required. In brief, these critics’ understanding is that the principles of beneficence, non-maleficence, autonomy, and justice are exhaustive in describing the ethics of AI, so while explicability may be important in AI development and implementation for medical purposes, it can only be so *derivatively* and not due to its status as a self-standing principle. Therefore, although Floridi and Cowls intend to tackle the problem of “principle proliferation” (2019, 2) in ethics guidelines by providing a simple five-principle set, this critique suggests that it is still too large as there is no justification for adding the principle of explicability.

A significant defense of this position comes from Ursin et al. (2022), who nevertheless recognize that AI-driven medicine brings special ethical challenges and potential harms, which might seem to support the addition of a further principle. They even compile some previous examples of healthcare specialisms adopting expanded sets of principles based on the classic bioethical four, such as the seven-principle public health ethics and ten-principle global health nursing frameworks. The technological complexity of AI in medicine, Ursin and co-authors highlight, means that the kind of disclosure necessary for informed consent and harm reduction no longer involves just two agents (as in the paradigmatic physician-patient relationship) but also technology developers. The resultant need for explicability implies “transparency”, which “involves mostly negative duties” not to withhold information, and the more stringent “explainability” and “demonstrability”, which “demand that information is made understandable to patients” (Ursin et al. 2022, 144). But Ursin et al.’s core argument, based on considering the views of professional bodies in radiology, is that such obligations can be derived from the four classic principles, inasmuch as they require harm reduction and informed consent. The principles of non-maleficence and beneficence require doctors to explain their reasoning to patients and encourage developers to produce explicable technologies as important mechanisms of human oversight intended to prevent AI-related harms. The principle of respecting autonomy equally entails the honest exchange of medical information in “a dialogue seeking mutual understanding,” such that patients can “understand their individual situation and how it was assessed” (Ursin et al. 2022, 151). Moreover, AI explicability, in Floridi et al.’s sense of ‘accountability’, seems to be necessitated by the principle of justice, which requires patients to be allowed to understand and appeal against healthcare outcomes on a fair and equal basis. If we can account for the impermissibility of inexplicable AI models without recourse to a fully-fledged *principle* of explicability, Ursin et al. conclude, why complicate our principlist model by granting explicability the independent weight that would involve?

At first glance, this reductionist position may be difficult to differentiate from Floridi et al.’s approach, given the subordinate role that the authors assign to explicability as an enabling principle in relation to the four taken directly from principlism in bioethics. To understand where their disagreement emerges, one must turn to the approach that reductionist critics have taken to the very idea of a ‘principle’ and the conceptual work it is meant to be doing in these accounts. According to Cortese et al. (2022, 4), “if explicability *always* comes together with other principles […] it is conditioned to them, and not a principle in itself”. This assertion rests on the notion that a moral principle is “a general norm” pertaining to certain “final ends”, an idea that can itself be supported from within the principlist tradition. Indeed, Beauchamp (1995, 182) asserts that “Childress and I use the term ‘principles’ to designate the most general normative standards of conduct”. On this understanding, when Floridi and his colleagues describe explicability as an ‘enabling principle’, they suggest that it is of the *most general* normative importance that AI be explicable *so that* the classic four bioethical principles are met. This appears contradictory to those who criticize the inclusion of explicability as a further principle enabling beneficence, non-maleficence, autonomy, and justice. In limiting explicability to an enabling role, one might say, Floridi et al. have conceded that explicability is simply what Cortese et al. call an “epistemic requirement” (2022, 2) for fulfilling the four principles, or perhaps an ethical one derived from them, while holding on to the misleading terminology of explicability as a ‘principle’ in itself.

## Re-establishing the principle of explicability

The idea that moral principles are to be found at the highest level of generality certainly complicates Floridi et al.’s picture of explicability within AI ethics, influential though it may be in the policy arena, and chimes with a certain strand of principlist thought. Yet it is far from the only position within the movement initiated by Beauchamp and Childress, whose own views have been subject to change, whether acknowledged or implicit, advanced in solo works or co-authored in new editions of their landmark text (Schöne-Seifert 2006). The specification and the balancing of principles are two areas of concern for those within the principlist tradition that are sometimes even thought to be the theory’s fatal flaws. However, the challenge of refining principlism goes beyond these two issues, with Launis (2009, 41) suggesting, for example, that “[t]he derivation of the principles, the number of principles, and the specification and balancing methods differ depending on the interpretation.“ To understand the derivation and enumeration problems, it is important to introduce and unpack Beauchamp and Childress’s picture of the four classic principles as ‘mid-level principles’. In the classic principlist picture, these principles appear as part of “an attempt to bypass intractable disagreements at the level of normative ethical theory and the resulting lack of agreement about how to proceed” (Keeling and Bellefleur 2016, 2). Thus, as Holm says their “moral force comes from the fact that the principle can be agreed upon as (almost) right by proponents of different moral theories” (1999, 59). Yet, Holm argues, Beachamp and Childress’s approach to the ‘mid-level’ has evolved to such an extent that by the fourth edition, “the authors have […] changed the way they derive the principles, and are proposing a radically different theory” (Holm 1999, 52). In the updated version of the theory, “midlevel principles are derived from common morality” and the intuitions associated with it, on Holm’s reading. Therefore, it seems that in the new principlism, an important aspect of the mid-level—namely, its mediation among moral theories—has been lost, and the principles appear to have transferred their derivation to the very general level of the common morality.

My intention here is not to insist on the earlier form of principlism over the later one; indeed, Beauchamp and Childress modified the source of their principles to the common morality after successive critiques and defenses of principlism that cannot be rehearsed here (Clouser 1995; Gert et al. 2000). On the contrary, my aim is to *widen* the understanding that operates in the explicability debate of what may count as a moral principle in the tradition of Beauchamp and Childress. What the varying interpretations of principlism show for present purposes is that explicability itself does not need to be immediately obvious at the most general level to function as a legitimate principle. Instead, it may receive some limited assent from a variety of normative theories as a way of mediating between their general schemas and practical guidance for moral decision-making. To see how this might look in our case, it is instructive to consider how new principles have previously been added to the traditional four-principle framework. Particularly relevant here is what DeMarco (2005, 101) calls “the mutuality principle”, which he proposes as a way of tackling the oft-cited problem of conflict among the four principles. This principle states that one ought to “establish the mutual enhancement of all basic moral values” (DeMarco 2005, 102) and, according to DeMarco, is needed in all cases where conflict between principles is likely. In terms of moral obligations, DeMarco believes that where principles threaten to conflict, an agent has a duty, flowing from the mutuality principle, not only to feel regret or compensate any victims after the fact but to make efforts to avoid the conflict altogether. This principle clearly plays a supportive—or to use our earlier terminology ‘enabling’—role when added to the classic four, which DeMarco believes to be justified by its adding to the overall coherence of the principlist framework in its application to moral scenarios. It is also “at a highly abstract level, like the other principles”, DeMarco asserts, yet “is in some sense about the other four principles, and so it is not derivable from them” (DeMarco 2005, 103).

My suggestion is that something similar can be said of explicability, which is ‘about’ the classic four principles insofar as it provides specific epistemic and ethical conditions for their fulfilment. In much the same way as DeMarco says mutuality is needed in bioethics to support the balancing of principles in the event of a conflict, explicability is able to mobilize the other ethical principles when we are examining any case of AI in medicine. By placing demands to build and use explicable models, it requires AI developers and medical practitioners to establish the conditions that mean the classic four principles can be applied and fulfilled. The principle of explicability is somewhat technical and certainly cannot be found at the highest level of generality where common-sense moral intuitions reside. Rather, its limited specificity—in contrast to both high-level moral theories and ground-level rules or guidance—is in fact what grants the principle of explicability its power as a mid-level moral principle. All plausible moral theories will agree that professionals must understand what they are working with to be held responsible for injustices, harms, autonomy-infringements, and so on (since ‘ought’ implies ‘can’) and that it would be reckless to use tools they knew they could not understand. Equally, the principle of explicability’s devotion to the use and development of AI models means that the novel and distinctive implications of AI in medicine are addressed before any of the classic four principles are lifted from bioethics and applied. Mediating between the two levels in this way is, according to the more established interpretation of principlism, precisely what makes explicability a principle necessary for the ethics of AI in medicine. Of course, this implies a hierarchy among the five principles of AI ethics, with explicability being subordinate to the others, but the four classic principles have been ranked before, in part to deal with perceived conflicts between them. Gillon is an especially well-known advocate of principlism in Britain who nevertheless explicitly assigns the principle of autonomy the status of “primus inter pares—first among equals” (2003, 310), just as critics of principlism accuse Beauchamp and Childress of doing surreptitiously (Callahan 2003).

## Anticipating an objection: stuck between principle overreach and a double standard?

The principle of explicability, then, enjoys a kind of justification that is not unlike that of the traditional principles of bioethics: it receives rough consensus from substantive moral theories while speaking directly to the practicalities of ethical decision-making. We can even see Floridi et al.’s work as a call to affirm this kind of enabling position as a strength in the case of explicability, rather a disqualifier from the privileged status of a moral principle. In response to my defense of explicability as an enabling principle, a staunch supporter of the four-principles approach might, if at all sympathetic, agree that it looks attractive when applied solely to AI-enabled medicine but argue that such a delimitation is unjustifiable given the black-box nature of human decision-making. The claim that AI models and human reasoners are equivalently opaque from the outside has been made frequently in recent scholarship, both with reference to human reasoners in general and clinicians specifically. Zerilli et al. (2019, 663) argue that “much human decision-making is fraught with transparency problems”, while London (2019, 18) claims that the opacity of ML approaches is “not radically different from routine aspects of medical decision-making.“

When prefaced with a statement of the principle of explicability, the parallel drawn between the inexplicability of human and AI decision-makers gives us the following syllogism with a troubling conclusion:

P1. If explicability is a principle, then any use of unintelligible, unaccountable decision-making in medicine violates principlism.

P2. Clinicians consistently use unintelligible, unaccountable decision-making in medicine.

C. Therefore, if explicability is a principle, then clinicians consistently violate principlism.

While it would be possible to accept this conclusion, it seems obvious that the principle of explicability could never be intended to apply to the whole of medicine. The most plausible rationale for this reaction, though rarely spelled out, seems to be that such an expansion would be unacceptably revisionist: ethicists throughout history, including principlists, have looked on everyday clinical decision-making as unproblematic, yet this novel principle of explicability wants us to think otherwise. Accepting a principle so broad would mean making demands on physicians well outside explicability’s original scope and doing so based on the use of AI alone, akin to an ethicist of dermatology making sweeping criticisms of doctors across specialisms because of a certain analysis of skin grafting. I wish to call this outcome ‘principle overreach’ and believe that its embrace can reasonably be rejected as a plausible option for the defender of explicability as a principle. Critics are therefore correct to focus on the matter of explicability’s limited application to AI in medicine, which they regard as setting an indefensible double standard for the equally opaque human and AI decision-making processes that may operate in medicine. If either P1 or P2 is false, however, the limited scope of the principle of explicability to AI in medicine can be justified. There would then be a normative reason why some black boxes in medicine are excluded from the explicability principle or else a material difference between the (in)explicability of AI models and clinicians such that the latter do not consistently unintelligible and unaccountable. While there are those who reject P1 by supporting the imposition of a normative double standard on AI and human decision-making (Günther & Kasirzadeh 2021), I will instead seek to use the remainder of this section to undermine their supposed descriptive equivalence.

At the core of the argument for P2 lies a skepticism about the capacity for humans to identify the reasons for their own decisions that draws on psychological research “[finding] a disconnect between human rationalizations and the factors that actually caused the actions so rationalized” (Buckner 2021, 32). This frequent inability on the part of human decision-makers to provide reasons reliably for our decisions arises out of the types of cognitive processes we tend to rely on, including “intuition, personal impression, and unarticulated hunches” (Zerilli 2019, 665). Yet, as Peters (2022, 8) argues, “the opacity of a human mental process … does not exclude it from also being a rational process based on conscious reasons” since the relevant reasons may, in the case of decision-making, be social norms that humans internalize in the process of gaining expertise in areas such as medicine. Scrutinizing the social environments of clinicians may allow us to access the reasons behind humans’ decisions, even if they cannot be offered by decision-makers themselves. This is not possible, however, in the case of AI decision-making, which involves important elements of human reasoning in practices of “defining features, pre-classifying training data, and adjusting thresholds and parameters” (Burrell 2016, 3) but is not reliably linked to “internalized, initially reflective and socially validated structures” (Peters 2022, 9).

Defenders of the equivalence between human and AI decision-makers tend to focus on their shared inability to present reasons for their decisions *themselves*; but in terms of intelligibility and accountability, it is difficult to tell why an explanation that we are given would be preferable to one that we find. Epistemically, our having access to knowledge about social norms that explain human decision-making processes seems to make them straightforwardly more intelligible than their AI counterparts. Morally, meanwhile, I admit that there is a certain tradition in political thought, reflected in the Rawlsian perspective mentioned above, that requires decision-makers to provide their reasons as a condition of accountability (Binns 2017). Yet one can also argue that there are times when only structural factors beyond the ken of individual human decision-makers, such as “widespread habits of thought, commonplace social practices, [and] compliance with formal or informal norms” (Himmelreich and Lim 2023) provide a sufficient explanation for the purposes of identifying and rectifying injustices. I conclude that any insistence that the reason for a decision must be offered by the decision-maker, when it is accessible by scrutinizing the wider environment influencing the process, stands in need of further defense, lest the defender of AI/human equivalence be accused of imposing a double standard of her own.

## Conclusion

This paper has attempted to fill a gap in the AI and medical ethics literature by offering a defense of the five-principle approach that incorporates both traditional principles of bioethics and a new principle tailored to the challenges raised by AI. In response to a rising tide of criticism directed at Floridi and colleagues’ approach that sees the addition of a principle of explicability as somehow unnecessary or incoherent, this paper has made two key points. First, the reductionist critique’s insistence on viewing moral principles as drawing justification at the highest level of abstraction ignores earlier versions of principlism that rest on the approximation of consensus from across moral theories. Second, an existing proposal for a ‘mutuality principle’ in addition to the four traditional principles can provide a model for the kind of justification that explicability receives due to enhancing the overall power of the principlist approach to AI in medicine. In response to a potential objection that draws a parallel between human and AI decision-making to suggest that the principle of explicability involves either overreach or a double standard, this paper has argued that it unfairly obscures the epistemic and moral potential of structurally explaining human decision-making.

When it comes to the development and implementation of AI in medicine, therefore, this paper supports the inclusion of a principle of explicability in order for it to play an enabling role in future ethical theories, codes, and frameworks. At this stage in the idea’s development, the prospects of translating the principle of explicability into specific recommendations and processes for AI are uncertain, as is generally the case for high-level theoretical propositions (Mittelstadt 2019; Seger 2022), and limited attention has been given to its cross-cultural applicability (Carman and Rosman 2020). However, an advantage of rooting our conception of explicability in the principlist tradition is that it can be debated in future research by drawing on existing empirical assessments of principlism in bioethics as a means of analyzing health technologies and establishing professional norms (Bosk 2010; Saarni et al. 2011). As well as furthering philosophical debate, therefore, I hope with this paper to encourage attempts to integrate and evaluate the principle of explicability as a complement to the standard principles of bioethics in the rapidly growing practice of AI-driven medicine.

## Footnotes

1 This paper uses ‘medical AI’ and ‘AI in medicine’ interchangeably to refer to all potential uses of AI for healthcare purposes.

2 It is worth noting that the language of AI *deciding*, rather than simply advising human decision-makers who have the final say, is contentious and has significant implications for the law and the patient-physician-relationship (de Bruijn et al. 2021; Lorenzini et al. 2023). However, without the space to delve into conceptual issues about the nature of decision-making, I follow Floridi et al. here in using this terminology.

## References

[CR1] Adamson, Greg. 2022. *Ethics and the explainable artificial intelligence (XAI) movement*. August. 10.36227/techrxiv.20439192.v1. TechRxiv.

[CR2] American Civil Liberties Union. 2023. Accountability in Artificial Intelligence. https://www.aclu.org/issues/racial-justice/accountability-in-artificial-intelligenceAccessed April 19.

[CR4] Beauchamp Tom L (1995). Principlism and its alleged competitors. Kennedy Institute of Ethics Journal.

[CR3] Beauchamp Tom L., Childress James F. (1979). Principles of biomedical ethics.

[CR5] Binns Reuben (2017). Algorithmic accountability and public reason. Philosophy & Technology.

[CR6] Bosk Charles L (2010). Bioethics, raw and cooked: extraordinary conflict and everyday practice. Journal of Health and Social Behavior.

[CR7] Buckner Cameron (2021). Black boxes, or unflattering mirrors? Comparative bias in the science of machine behavior. The British Journal for the Philosophy of Science.

[CR8] Burrell Jenna (2016). How the machine thinks: understanding opacity in machine learning algorithms. Big Data & Society.

[CR9] Callahan Daniel (2003). Principlism and communitarianism. Journal of Medical Ethics.

[CR10] Carman, Mary, and Benjamin Rosman. 2020. Applying a principle of explicability to AI research in Africa: should we do it? *Ethics and Information Technology* 23. 10.1007/s10676-020-09534-2.

[CR11] Caruana, Rich, Yin Lou, Johannes Gehrke, Paul Koch, Marc Sturm, and Noémie Elhadad. 2015. Intelligible models for HealthCare: Predicting pneumonia risk and hospital 30-Day readmission. In Proceedings of the 21th ACM SIGKDD International Conference on Knowledge Discovery and Data Mining, 1721–1730. Sydney, NSW, Australia: Association for Computing Machinery. 10.1145/2783258.2788613.

[CR12] Clouser K Danner, Danner (1995). Common morality as an alternative to principlism. Kennedy Institute of Ethics Journal.

[CR13] Cortese, João Figueiredo Nobre Brito, Fabio Gagliardi Cozman, Marcos Paulo Lucca‑Silveira, and Adriano Figueiredo Bechara. 2022. Should explainability be a fifth ethical principle in AI ethics? AI and Ethics. 10.1007/s43681-022-00152-w.

[CR14] de Bruijn Hans, Warnier Martijn, Janssen Marijn (2021). The perils and pitfalls of explainable AI: strategies for explaining algorithmic decision-making. Government Information Quarterly.

[CR15] DeMarco Joseph P (2005). Principlism and moral dilemmas: a new principle. Journal of Medical Ethics.

[CR16] European Commission Directorate-General for Technology. 2019. Ethics guidelines for trustworthy AI. Publications Office of the European Union. Publications Office. 10.2759/346720.

[CR17] Floridi Luciano (2002). Information ethics. Philosophy in the Contemporary World.

[CR18] Floridi, Luciano, and Josh Cowls. 2019. A unified framework of five principles for AI in society. *Harvard Data Science Review* 1. 10.1162/99608f92.8cd550d1.

[CR19] Floridi Luciano, Cowls Josh, Beltrametti Monica, Chatila Raja, Chazerand Patrice, Dignum Virginia, Luetge Christoph (2018). AI4People—an ethical framework for a good AI society: Opportunities, risks, principles, and recommendations. Minds and Machines.

[CR20] Gert Bernard, Culver Charles M, Danner Clouser K (2000). Common morality versus specified principlism: reply to Richardson. The Journal of Medicine and Philosophy.

[CR21] Gillon Raanan (2003). Ethics needs principles—four can encompass the rest—and respect for autonomy should be "first among equals". Journal of Medical Ethics.

[CR22] Günther, Mario, and Atoosa Kasirzadeh. 2021. Algorithmic and human decision making: for a double standard of transparency. *AI & Society* 37. 10.1007/s00146-021-01200-5.

[CR23] Hedlund, Maria, and Erik Persson. 2022. Expert responsibility in AI development. AI & Society. 10.1007/s00146-022-01498-9.

[CR24] Himmelreich, Johannes, and Désirée Lim. 2023. AI and structural injustice: Foundations for equity, values, and responsibility. In *The Oxford handbook of AI governance*, ed. Justin B. Bullock, Yu-Che Chen, Johannes Himmelreich, Valerie M. Hudson, Anton Korinek, Matthew M. Young, and Baobao Zhang. Oxford: Oxford University Press. 10.1093/oxfordhb/9780197579329.013.13.

[CR25] Holm Søren (1999). Principles of health care ethics: solution or problem? In genes and morality.

[CR26] Information Commissioner’s Office, and Alan Turing Institute. 2020. What goes into an explanation? https://ico.org.uk/for-organisations/guide-to-data-protection/key-dp-themes/explaining-decisions-made-with-artificial-intelligence/part-1-the-basics-of-explaining-ai/what-goes-into-an-explanation/. July 20.

[CR27] Jobin Anna, Ienca Marcello, Vayena Effy (2019). The global landscape of AI ethics guidelines. Nature Machine Intelligence.

[CR28] Keeling Michael, Bellefleur Olivier (2016). Principlism and frameworks in public health ethics.

[CR30] Launis Veikko (2009). The unbearable lightness of bioethical principles. Cutting through the surface: philosophical approaches to bioethics.

[CR31] Lipton Zachary C (2018). The mythos of model interpretability. Communications of the ACM.

[CR32] Loi, Michele, Christoph Heitz, and Markus Christen. 2020. A comparative assessment and synthesis of twenty ethics codes on AI and big data. In 2020 7th Swiss Conference on Data Science, 41–46. 10.1109/SDS49233.2020.00015.

[CR34] London Alex J (2019). Artificial intelligence and black-box medical decisions: Accuracy versus explainability. Hastings Center Report.

[CR33] Lorenzini, Giorgia, Laura Arbelaez Ossa, David Martin Shaw, and Bernice Simone Elger. 2023. Artificial intelligence and the doctor–patient relationship expanding the paradigm of shared decision making. *Bioethics* 37. Wiley-Blackwell. 10.1111/bioe.13158. 10.1111/bioe.13158 36964989

[CR35] Maclure Jocelyn (2021). AI, explainability and public reason: the argument from the limitations of the human mind. Minds and Machines.

[CR36] Mitcham Carl (1994). Thinking through technology: the path between engineering and philosophy.

[CR37] Mittelstadt Brent (2019). Principles alone cannot guarantee ethical AI. Nature Machine Intelligence.

[CR38] Morley, Jessica, Luciano Floridi, Libby Kinsey, and Anat Elhalal. 2019. From what to how: an initial review of publicly available AI ethics tools, methods and research to translate principles into practices. *Science and Engineering Ethics* 26. 10.1007/s11948-019-00165-5. 10.1007/s11948-019-00165-5 PMC7417387 31828533

[CR39] Páez Andrés (2019). The pragmatic turn in explainable artificial intelligence (XAI). Minds and Machines.

[CR40] Peters Uwe (2022). Explainable AI lacks regulative reasons: why AI and human decision-making are not equally opaque. AI and Ethics.

[CR41] Saarni Samuli I, Braunack-Mayer Annette, Hofmann Bjørn, Gert Jan van der Wilt (2011). Different methods for ethical analysis in health technology assessment: an empirical study. International Journal of Technology Assessment in Health Care.

[CR42] Schöne-Seifert Bettina (2006). Danger and merits of principilism: Meta-theoretical reflections on the Beauchamp/Childress-approach to biomedical ethics. Bioethics in Cultural Contexts: reflections on methods and finitude.

[CR43] Schuck Peter H (1994). Rethinking informed consent. The Yale Law Journal.

[CR44] Seger, Elizabeth. 2022. In defence of principlism in AI ethics and governance. *Philosophy & Technology* 35. 10.1007/s13347-022-00538-y.

[CR46] Ursin Frank, Timmermann Cristian, Steger Florian (2021). Explicability of artificial intelligence in radiology: is a fifth bioethical principle conceptually necessary?. Bioethics.

[CR45] Ursin Frank, Lindner Felix, Ropinski Timo, Salloch Sabine, Timmermann Cristian (2023). Levels of explicability for medical artificial intelligence: what do we normatively need and what can we technically reach?. Ethik in der Medizin.

[CR47] Zerilli John, Knott Alistair, Maclaurin James, Gavaghan Colin (2018). Transparency in algorithmic and human decision-making: is there a double standard?. Philosophy & Technology.

